# Views of Hong Kong Chinese medicine practitioners on the application of the “Chinese Medicine Anti-epidemic Plans” prepared by the Chinese medicine expert group of central authorities: a focus group study

**DOI:** 10.1186/s12906-024-04469-3

**Published:** 2024-05-04

**Authors:** Shu Cheng Chen, Wing Fai Yeung, Hui Lin Cheng, Man Ho Li, Yuen Shan Ho

**Affiliations:** School of Nursing, The Hong Kong Polytechnic University, 11 Yuk Choi Road, Hung Hom, Kowloon, Hong Kong China

**Keywords:** Chinese medicine, Anti-epidemic plans, COVID-19 pandemic, CM practitioner, Hong Kong, Focus group, Qualitative study

## Abstract

**Background:**

Drawing on the extensive utilization of traditional Chinese medicine (TCM) to combat COVID-19 in Mainland China, experts designed a series of TCM anti-epidemic strategies. This study aims to understand Hong Kong CM practitioners’ application of and opinions on the “Chinese Medicine Anti-epidemic Plans.”

**Methods:**

Online focus group interviews were conducted, and purposive sampling was employed to invite 22 CM practitioners to voluntarily participate in three interview sessions. The interviews were audio recorded, then transcribed verbatim. The transcripts were analyzed using template analysis.

**Results:**

Three themes were derived: (1) facilitators of the “Chinese Medicine Anti-epidemic Plans,” (2) barriers of the “Chinese Medicine Anti-epidemic Plans,” and (3) expectations on improving the “Chinese Medicine Anti-epidemic Plans.” The participants could obtain relevant information from various sources, which highlights the value of the plans for TCM medicinal cuisine and non-pharmacologic therapies and guiding junior CM practitioners, supplementing Western medicine interventions, and managing Chinese herb reserves in clinics. However, the barriers included the lack of a specialized platform for timely information release, defective plan content, limited reference value to experienced CM practitioners, and lack of applicability to Hong Kong. The expectations of the CM practitioners for improving the plans were identified based on the barriers.

**Conclusions:**

To enhance the implementation of the anti-epidemic plans, CM practitioners in Hong Kong expect to utilize a specific CM platform and refine the plans to ensure that they are realistic, focused, comprehensive, and tailored to the local context.

**Supplementary Information:**

The online version contains supplementary material available at 10.1186/s12906-024-04469-3.

## Background

The COVID-19 pandemic, caused by the novel coronavirus SARS-CoV-2, led to a global health crisis of unprecedented magnitude [1]. As the virus spread rapidly across borders, infecting millions and posing a threat to healthcare systems worldwide [2, 3], various medical approaches were explored to mitigate its impact [4–6]. Traditional Chinese medicine (TCM), with its rich history and holistic approach to healthcare, emerged as a valuable resource for combating diseases [7, 8]. Rooted in centuries of traditional knowledge and practices, TCM encompasses a range of modalities, including the use of Chinese herbs, acupuncture, moxibustion, and auricular therapy [9]. Such therapeutic techniques have been extensively examined and applied in China, suggesting their potential in managing infectious diseases [10]. In the face of the COVID-19 pandemic, experts in TCM formulated and adopted specific anti-epidemic strategies based on their experience and the clinical plans implemented in Mainland China, to provide valuable insights and instructive recommendations for the effective utilization of TCM across populations and strata [7]. A comprehensive and integrative approach can be created to address the diverse needs of individuals affected by the pandemic by incorporating TCM into the existing healthcare framework [7].

Drawing on the extensive utilization of TCM in Mainland China to combat diseases, and considering the pre-existing clinical plans employed in Hong Kong, a group of experts formulated a series of TCM anti-epidemic strategies that are intended for practical implementation [11]. Such plans can serve as instructive guidelines for the CM domain and public, which outline the appropriate utilization of CM across strata and target populations. Specifically, the guidelines cater to close contacts, secondary close contacts, asymptomatic individuals, individuals displaying mild symptoms, patients receiving care in isolation facilities, and those in the process of recovery. The “Chinese Medicine Anti-epidemic Plans” include “Recommendations on the Use of Proprietary Chinese Medicines at Home for COVID-19 (Pilot Version),” the “COVID-19 Chinese Medicine Treatment Plan (Pilot Version),” and “Guidance and Recommendations on Chinese Medicine Rehabilitation During COVID-19 Recovery Stage (Pilot Version)” [12]. To gain a deep understanding of Hong Kong CM practitioners’ application of and opinions on the guidelines, we designed and conducted focus group interviews. Many proprietary CMs in China are not available in Hong Kong; thus, we focused on the “COVID-19 Chinese Medicine Treatment Plan (Pilot Version)” and “Guidance and Recommendations on Chinese Medicine Rehabilitation During COVID-19 Recovery Stage (Pilot Version).”

## Methods

### Ethical approval

was obtained from the Institutional Review Board of the Hong Kong Polytechnic University (HSEARS20220428008). The Consolidated Criteria for Reporting Qualitative Research checklist was used to guide the reporting of this study [13].

### Aim

This study aimed to explore and collect information on Hong Kong CM practitioners’ application experience in and opinions on the “Chinese Medicine Anti-epidemic Plans,” specifically the “COVID-19 Chinese Medicine Treatment Plan (Pilot Version)” and “Guidance and Recommendations on Chinese Medicine Rehabilitation During COVID-19 Recovery Stage (Pilot Version).”

### Design

This study employed a qualitative approach, that is, semi-structured focus group discussions. The research methodology will enable researchers to gain an in-depth understanding of specific topics and allow the active participation of the respondents, who are expected to contribute their expertise and experience.

### Setting and sampling

All the respondents, who were registered CM practitioners, participated in the online focus group interviews. The participants were recruited from three local CM practitioner associations (i.e., Sin-Hua Herbalists & Herb Dealers’ Promotion Society, Hong Kong Baptist University School of Chinese Medicine Alumni Association, and the Chinese Medicine Alumni Association of CUHK) from July to September 2022. Purposeful sampling was conducted for the sample selection, and the CM practitioners who were willing to participate in the interviews were invited to join the study [14]. The CM practitioners who were included in the qualitative study (1) were registered TCM practitioners in Hong Kong and (2) had clinical experience in the diagnosis and treatment of at least 30 COVID-19 patients during the fifth wave of the COVID-19 pandemic in Hong Kong. Written informed consent was obtained from all the participants, and each focus group interview was led by the principal researcher (YSH) as the moderator, with a research assistant (MHL) as the assistant moderator. All the focus group interviews were conducted online via Zoom as a social-distancing measure during the COVID-19 pandemic and to prevent the spread of the virus.

### Data collection

The focus group interviews were moderated in accordance with a practical guide to focus groups written by Krueger et al. [15]. A semi-structured interview guide was developed and revised on the basis of experts’ comments, including two TCM practitioners (YSH [PhD] and WFY [PhD]) and a qualitative researcher (HLC [PhD]). The guide consisted of four open-ended questions (Table 1). The moderator (YSH) establish a strong rapport with the participants, because some of them were alumni, and others were from the same local TCM associations. The moderator was an experienced Hong Kong CM practitioner with strong research expertise in TCM intervention and trained by an experienced qualitative researcher (HLC). In each interview session, the moderator introduced herself, explained the purpose and process of the meeting, highlighted the rules on confidentiality, explained the questions, constantly provided prompts and pauses to facilitate interaction between the group members without expressing any value judgments, and ensured that the participants were focused on the discussion. The assistant moderator (MHL) took notes throughout the discussion and operated the recording equipment during the interviews. Participation was voluntary. No other individuals besides the participants and researchers were present during the interviews. All the qualitative interviews were audio recorded. For confidentiality, all the participants were asked to turn off all audio and video devices to provide a natural and safe atmosphere. Each participant was offered a HKD 200 cash voucher for their participation.

**Table 1:** Questions for the semi-structured interview

1. Before participating in this focus group discussion or during the fifth wave of the pandemic, have you heard or come across the two clinical application plans developed by experts from the Mainland? (If yes, how did you come across these application plans?)	
2. In summary, do you think these two plans have any reference value for your treatment of COVID-19 (including treatment and rehabilitation)? Why?	
3. If the next COVID-19 patient seeks treatment, would you refer to the above plans for your treatment? Why or why not?	
4. What content should be added to enhance the reference value of the “Clinical Application Plan of Traditional Chinese Medicine against Epidemic”?	

### Data analysis

All the interviews were audio recorded, then transcribed verbatim in traditional Chinese before the data analysis. Each participant was renamed by using a code to ensure confidentiality. All the study-related documents and transcripts were deidentified, and all the audio files were destroyed after the completion of the transcription. Descriptive characteristics were used for the demographic data to provide the sample profile. The transcripts were analyzed using template analysis, which is a thematic analysis style that stresses the use of hierarchical coding [16, 17]. Template analysis can be roughly divided into several steps. (a) The transcripts were read to determine the a priori themes and conduct preliminary coding. (b) The initial template was defined. (c) All the data sets were examined systematically to modify the template by inserting, deleting, or merging contents, if necessary. (d) The template for the full data set was finalized. The assistant moderator (MHL) and first author (SCC) transcribed the interviews and coded the data. In addition, the two researchers independently read all the interview transcripts to gain an in-depth understanding of the data, completed the data immersion process, and performed independent coding by writing the words or codes from each interview that seemed to capture important thoughts or concepts. Then, the researchers compared the codes, finalized the initial coding scheme, and sorted the codes into emergent themes that represented the key findings from the interviews. The researchers resolved discrepancies by consulting the principal researcher (YSH). The other authors provided feedback on the data analyses. Coding management was facilitated using MS Word [18, 19].

### Trustworthiness

Several strategies were used to determine the trustworthiness of the study findings based on the four criteria created by Lincoln: credibility, dependability, conformability, and transferability [20, 21]. (1) Credibility: The interview proposal was revised during two group meetings. The researchers were equipped with the required knowledge and expertise to perform their responsibilities. To reduce researcher bias, all the research team members provided feedback on the analyses and study findings. (2) Dependability: The findings were presented to the participants for comments and confirmation. The coding structure was validated by another researcher (SCC), then revalidated by the corresponding author (YSH) by reviewing the original transcript. (3) Confirmability: The transcripts were given to the participants for feedback. Therefore, negative case analysis was adopted to enhance the trustworthiness of the qualitative data. (4) Transferability: The researchers determined the richness of the data by applying saturation theory [22] and continued to gather data until new information was nearly exhausted by the time five sessions were reached [23].

## Results

The focus group interviews were conducted in Cantonese between July and September 2022. A total of 31 Hong Kong CM practitioners were invited to participate in the study, and 22 accepted the invitation. Five focus group interviews were conducted. The interviews had a mean duration of 60.4 min and ranged from 37 min to 110 min. The number of participants in each group ranged from 3 to 6. Most of the participants in each group were acquainted with one another. Data saturation was applied to guide the data collection [22], with themes and subthemes that were established in the first three focus group interviews and enriched after the fifth focus group interview.

### Sample profile

A total of 22 Hong Kong CM practitioners (9 women [41.0%] and 13 men [59.0%]) participated in the five focus group interview sessions. The mean age of the participants was 37.3 years (SD = 4.8). All the CM practitioners had at least a bachelor’s degree, 7 (31.8%) had a doctoral degree, and 12 (54.5%) had a master’s degree. The participants’ demographic information is presented in Table 2.

**Table 2:** Demographic Characteristics of Participants Interviewed (*N* = 22)

Characteristics	All participants (*N* = 22)	Session 1 (*N* = 6)	Session 2 (*N* = 5)	Session 3 (*N* = 5)	Session 4 (*N* = 3)	Session 5 (*N* = 3)
Age, mean (SD)	37.3 (4.8)	35.8 (4.3)	36.7 (6.6)	38.6 (2.3)	36 (7.2)	40.3 (4.0)
Gender, no. (%)						
Male	13 (59.1)	3 (50)	2 (40)	4 (80)	3 (100)	1 (33.3)
Female	9 (40.9)	3 (50)	3 (60)	1 (20)	0	2 (66.6)
Highest Educational Degree, no. (%)						
Bachelor Degree	3 (13.6%)	1 (16.7)	1 (20)	1 (20)	0 (0)	0 (0)
Master Degree	12 (54.5%)	5 (83.3)	3 (60)	1 (20)	2 (66.7)	1 (33.3)
PhD Degree	7 (31.8%)	0	1 (20)	3 (60)	1 (33.3)	2 (66.7)
Years of clinical practice, mean (SD)	12.2 (4.4)	10.3 (4.1)	13 (6.1)	12.8 (2.2)	12.7 (6.8)	13 (4.6)

### Major themes

In terms of the CM practitioners’ experience with the “Chinese Medicine Anti-epidemic Plans,” three themes were identified from the data: (1) facilitators of the “Chinese Medicine Anti-epidemic Plans,” (2) barriers of the “Chinese Medicine Anti-epidemic Plans,” and (3) expectations on improving the “Chinese Medicine Anti-epidemic Plans.” The specific subthemes under each theme were described, and Table 3 presents the code structure.

**Table 3:** Code structures

Themes	Sub-themes	Code Unites
Facilitators of the “Chinese Medicine Anti-epidemic Plans”	• Diversified information channels	• Traditional Chinese medicine groups, social media, work, non-profit organizations, hospital authority, training, and reference to the mainland version
	• Reference value of the Plans	• TCM medicinal cuisine, acupuncture and moxibustion, non-pharmacological interventions, and others have a reference value
	• The value to patients	• It holds reference value for the application in the Asia World-Expo Isolation ward (Asia World-Expo) • Effective for inpatients, patients with comorbid chronic diseases, and patients with deficiency syndromes
	• The value to TCM physicians	• The guideline is useful for TCM practitioner with limited clinical experience • Prepare more TCM resources for practitioners address the demand and complement Western medicine therapy • During the epidemic, it can be used diagnosis to guide the reserve of traditional Chinese medicine and prescription ideas
Barriers of the “Chinese Medicine Anti-epidemic Plans”	• Issues on plan release	• No official release channel, difficult to search, and uncertain version accuracy • Released too late, pass the peak of the epidemic
	• Defects in the content of the plan	• Confusion in the management of prevention/treatment/prognosis, incomplete content • The treatment methods have limitations: Collecting the ingredients for TCM medicinal cuisine is difficult, and the feasibility of non-pharmacological interventions (e.g., acupuncture and moxibustion, massage, TCM medicinal cuisine, emotional therapy and respiratory therapy) is poor • Limited use for outpatient clients and sequelae
	• Low reference value to TCM practitioners	• When it was released, TCM practitioners had accumulated experience and had not fully agreed with the prescriptions in the plan.
	• Unsuitability for local application	• Lack of local typical syndrome types in the plan (e.g., damp-heat syndrome) • Majority of the local cases involve outpatient visits
Expectations on improving the “Chinese Medicine Anti-epidemic Plans”	• Plan release mechanism improvement	• Suggested establishing an official organization for disseminating information • A Cantonese version is needed • Provide forward-looking information and continuously update the plans
	• Plan content improvement	• Supplement diagnosis and treatment contents (etiology and pathogenesis, diagnose and medication, provide detailed acupuncture and moxibustion protocol, clear visual teaching of Qigong and other therapies) • Provide more targeted medication recommendations based on known clinical data • Supplement information sources • Summarizing and sharing of clinical cases
	• Enrichment of information sources for plan development	• Organize local TCM practitioners to rapidly gather diagnosis and treatment experience and characteristics of the disease during the outbreak • Establish a communication platform to collect all information and opinions • The framework of the plan should be based on the clinical treatment experience, and reference to expert suggestions to enhance the treatment approach
	• Plan localization	• Tailor treatment according to three categories of etiological factors, refining the syndrome differentiation, diagnosis and treatment content to adapt to the local climate and seasonal variations • Involve local TCM teams in formulating the plan

#### Facilitators of “Chinese medicine anti-epidemic plans”

This theme consisted of four subthemes: (a) diversified information channels, (b) reference value of the plans, (c) the value to patients, and (d) the value to TCM practitioners.

##### Diversified information channels

All the participants mentioned that they had read or heard about the documents, and their ways of obtaining information varied, such as from TCM groups, social media, the workplace, nongovernment organizations, hospital authorities, peer exchanges, and TCM training. A participant (P1) shared that he obtained information on the documents from *“some TCM groups or classmate groups shared [the documents] with everyone.”* Another participant (P2) mentioned, *“I remember I hearing about that on TV news and I had seen it in the association.”*

##### Reference value of the plans

Most of the participants believed that the reference value of the “COVID-19 Chinese Medicine Treatment Plan (Pilot Version)” focused on TCM medicinal cuisine and non-pharmacologic therapies (e.g., acupuncture and acupressure). A participant (P11) said, *“Rehabilitation guidelines have their benefits, such as [for a] TCM medicinal cuisine. If patients have any inquiries, then you can answer them by referring to the guidelines.”* According to another participant (P5), *“Acupuncture and moxibustion interventions are described in detail instead, specific acupuncture points and duration are covered, massage therapy techniques are also included ”*.

##### The value to patients

Most of the CM practitioners affirmed the value of the “Guidance and Recommendations on Chinese Medicine Rehabilitation During COVID-19 Recovery Stage (Pilot Version)” to inpatients and patients with chronic underlying diseases or deficiency syndromes. One of the participants (P16) depicted the target users of CM prescriptions in the guidelines: “*They were sent from hospitals to AsiaWorld-Expo Asia World-Expo(a quarantine center operated by the Hong Kong Government). Most are old, with chronic diseases and deficiency syndromes. I think that the prescription is really useful for elderly people. They don’t cough much and their symptoms are related to comorbid chronic diseases. Most cases have recovered from illness after taking traditional Chinese medicine.*” One participant (P15) suggested the application of the guidelines in quarantine centers in Hong Kong: “*It was used frequently in AsiaWorld-Expo Asia World-Expo.*”

##### The value to TCM practitioners

The participants narrated the value of the two documents to Hong Kong CM practitioners from several aspects, including guiding the functions of new CM practitioners with little clinical experience, complementing Western medicine interventions, and managing Chinese herb reserves and prescription formulation. A participant (P21) agreed with the reference value of the documents to junior CM practitioners but disagreed with their value to experienced CM practitioners: “*For fresh graduates TCM practitioners believe it has a reference value, while highly experienced ones think it has none.*” A participant (P16) believed that the documents may help doctors use CM interventions to complement Western medicine: “*[For some COVID-19 symptoms], Western medicine can’t do much, there were no Covid-19 antivirals at that time…there is really no medicine to treat him. Just take these traditional Chinese medicines.*” Another participant (P7) mentioned that the documents could guide CM practitioners’ prescription ideas and reserves of different CMs in hospitals and clinics: “*After looking through this material, I may remind myself that such people (patients) exist. I may not have made a prescription for a pure ginseng decoction before. Should I prepare some Codonopsis pilosula (similar effect to ginseng) in the clinic first? Compared to the one released by the National Health Commission, this (Hong Kong) version may provide more insight in these areas.*”

#### Barriers of the “Chinese medicine anti-epidemic plans”

The barriers to the application of the anti-epidemic plans included five subthemes: (a) issues on plan release, (b) defects in the content of the plans, (c) low reference value to TCM practitioners, and (d) unsuitable for local application.

##### Issues on plan release

Despite all the channels through which the participants could obtain information on the plans, they all believed that the dissemination of the plans was problematic. The main problem was the lack of a specialized platform for CM practitioners in Hong Kong to get official guidance or information on the plans. A participant (P7) expressed this point, as follows: “*I know from the news that there would be a plan, but it is difficult to search, no matter which channel you used. We currently do not have an official channel for traditional Chinese medicine.*” Another problem was the late release of the documents, which missed the peak of the pandemic. “*I feel that if it were released earlier, with this version, I could accept it. Because at that time, when everyone started their clinical work, we had no chance to see these patients. This plan tells us what we will encounter, so it is all right. However, it was released in April, and the peak was over*” (P15).

##### Defects in the content of plans

The participants generally believed that the content of the plans was defective in several respects. First, the intervention methods occasionally crossed the broad line between prevention, treatment, and prognosis, which were confusing. “*I don’t know why in the industry there is often confusion between prevention, treatment and post-treatment. Why does one prescription, initially meant for prevention, turned into that for post-treatment? I think this is a big issue*” (P17). Second, the feasibility of TCM medicinal cuisine and non-pharmacologic therapies was limited, and preparations for all the materials needed for TCM medicinal cuisine therapy and manipulations of external therapies (e.g., acupuncture and *tuina*) were complicated. A CM practitioner (P12) complained, “*In addition, they (the suggested plans) are very complicated. Siraitia grosvenorii, ficus carica, asian pear, orange, radish, and olives should be all included, which is difficult. Some of them are complicated. I won’t do moxibustion and massage, so I won’t refer to the plan*.” Third, the effect of the plans on the sequelae of the pandemic was limited. A participant (P16) pointed out: “*Many patients who suffered from COVID-19 have different diseases (symptoms), these Chinese medicine prescriptions are useless, for those with emotional problems, these prescriptions may not help.*”

##### Low reference value to TCM practitioners

Many of the participants doubted the reference value of the plans to experienced CM practitioners, though they may provide some useful information to new CM practitioners. CM practitioners may have accumulated considerable experience by the time the plans were released, and the participants did not completely agree with the prescriptions in the plans. “*After the clinical guidelines were released, we had already conducted consultation with a lot of patients, the variant was Omicron, and we had basically mastered the entire pattern. So, the subsequent information didn’t hold much reference value for us.*” (P1).

##### Unsuitable for local application

The participants believed that the plans were not fully suitable for Hong Kong. On the one hand, the plans lacked typical local TCM patterns, such as the dampness–heat pattern. A participant (P12) said, “*It does not cover the therapy for the late-stage damp–heat diseases and warm-heat diseases. There is no content related to the warm-heat disease.*” On the other hand, outpatients accounted for the vast majority of the individuals who sought CM treatment, which differed from the mainland. Therefore, the feasibility of the plans was considerably compromised. “*I understand all the contents in the COVID-19 guidelines, but it is difficult to implement in Hong Kong due to insufficient conditions. Most of the cases we have are outpatient visits (outpatient visits refer to patients who are not isolated in facilities such as the AsiaWorld-Expo or Mobile Cabin Facilities Asia World-Expo)*” (P9).

#### Expectations on improving the “Chinese medicine anti-epidemic plans”

The expectations of the CM practitioners included three subthemes: (a) plan release mechanism improvement, (b) plan content improvement, (c) enrichment of information sources for plan development, and (d) plan localization.

##### Plan release mechanism improvement

The participants proposed several ideas for improving the mechanism for releasing the plans. Most of the CM practitioners believed that an official institution or sector was needed for the dissemination of CM-related information. One participant (P22) mentioned that Hong Kong could learn from the mainland regarding this aspect: “*In mainland China, the National Health Commission and State Administration of Traditional Chinese Medicine are responsible for jointly organizing experts to formulate these diagnosis and treatment plans, which are then publicly released. Therefore, a similar institution should be established in Hong Kong.*” Another participant (P18) expected the creation of a local version of the plans, as the pronunciation of some words is different between Mandarin and Cantonese: “*For respiratory therapy, shall it be practiced using Mandarin pronunciation? If it is pronounced in Mandarin, then it is different from our Cantonese pronunciation. Thus, do we need to do a Cantonese version?*” Pronunciation is essential for respiratory therapy, which involves patients’ self-practice and training of their vocal resonance chamber to improve their breathing. A few of the participants expressed that the plans should be timely and updated continuously to provide forward-looking information. One participant (P15) illustrated the need for quarterly updates: “*There should be an organization responsible for updating the information for doctors regularly (e.g., quarterly updates).*”

##### Plan content improvement

All the participants expressed their expectations on the improvement of the content of the “Chinese Medicine Anti-epidemic Plans.” For supplementary diagnosis and treatment, expectations in three areas were expressed, that is, disease symptoms and transmission, the coordination mode of CM and Western medicine, and the refinement of the content of nondrug treatment. A participant (P3) hoped to learn the difference between COVID-19 symptoms and common cold symptoms: “*I would like to know how those symptoms differ from the symptoms I usually see or how they differ from the cold symptoms I have seen before.*” Another participant (P22) emphasized its significance for screening critical symptoms: “*Pathogenesis, disease mechanisms, and identifying high-risk groups, so that doctors know which are critical symptoms and how to handle them accordingly.*” One of the participants (P5) believed that CM practitioners should know how to combine the CM plans with Western medicine: “*Add more content about Western medicine, including the intervention time of TCM. At that time, I could imagine that the combination of Chinese and Western medicines would be even better. This plan then will have great reference value, such as what kind of dialectics and what medicine to use.* ” She (P5) also expressed that additional information should be provided in the section on acupuncture: “*For acupuncture, there is no distinction between main acupoints, and there is no selection of acupoints based on syndrome differentiation. I believe that it can be written in more detail, and there are some aspects that I don’t know why they do it in this way?*” Besides the above expectations on diagnosis and treatment, most of the participants strongly recommended the provision of targeted and specific treatment plans for different user groups. “*Write down how to use Western and traditional Chinese medicine in parallel, and then observe the clinical performance of [the] patients. In this way, there is a clinical reference value. Medication treatment should be more clear, precise, and targeted; We cannot always generalize*” (P9). Another participant (P22) said, “*If a plan is issued, it may need to be divided into different directions. One is targeting the community, which can be accessed earlier. The other one is for various centers (isolation facilities).*” Some of the CM practitioners hoped for experts to supplement the documents with references. According to one participant (P8), “*The clinical protocol should have references. If there are references, everyone can look them up for further review. Maybe this could be much better?*” Case reports during a COVID-19 outbreak were also expected to be summarized and added to the documents for experience sharing among CM practitioners. A participant (P2) expressed, “*I think sharing actual cases, has more practical utility, and without pulse taking and tongue inspection, how do they prescribe medication and diagnose? I think these experiences are useful, but it may be challenging to incorporate them into the guidelines.*”

##### Enrichment of information sources for plan development

The participants expected the information sources of the documents to be enriched in three aspects. First, local institutions should rapidly collect information on treatment experiences and epidemic characteristics to summarize the disease patterns. “*Because nobody knows the characteristics and pattern of the disease in the human body, everyone is exploring. If you have the information, write it down*” (P1). Second, a communication platform should be set up in Hong Kong for CM practitioners to pool their ideas. “*Experience can be presented on a platform. Those who have less experience can gradually learn from more experienced TCM practitioners. This way, when others are working, there will be new experiences. This will not only help quickly establish a systematic framework, but it also adds reference value*” (P5). Third, the plans should originate from clinical treatment experiences and refer to the diagnosis and treatment ideas of experts from different folk schools. “Different schools of thought, *for example, those who use classical prescriptions, may have a different perspective from other TCM practitioners. There will be more diverse reference ideas and elements. I think this is better when compared to the current textbook-style or syndrome-based approach*” (P4).

##### Plan localization

Most of the participants challenged the localization of the plans and presented two ways to localize the plans. On the one hand, the plans should be formulated in accordance with the locality, time, and persons involved. One participant (P17) explained this point: “*I believe that for the same viral pathogen, there should be variations based on different seasons and weather conditions.*” On the other hand, local CM practitioner groups in Hong Kong should be involved in the development of the plans. One participant (P15) emphasized its importance: “*You need frontline TCM experts from our region. You definitely won’t have enough knowledgeable understanding of our situation compared to local TCM practitioners who have been practicing here for over a decade.*”

## Discussion

This focus group interview study is the first to comprehensively explore the perspectives of Hong Kong CM practitioners on the “Chinese Medicine Anti-epidemic Plans.” Focus group interviews can enable the thorough exploration of research topics by capturing the diverse perspectives of the participants, resulting in detailed and comprehensive data for a nuanced understanding of their ideas and experiences [24]. The group format can foster dynamic discussions, stimulating idea generation, novel insights, and the expression of perspectives that may be missed in individual interviews. Such interactions can enhance our understanding of complex social phenomena [2]. The qualitative data demonstrate the Hong Kong CM practitioners’ views on the “Chinese Medicine Anti-epidemic Plans,” with three themes: facilitators, barriers, and expectations. The participants can obtain relevant information from various sources, highlighting the value of the plans for TCM medicinal cuisine and non-pharmacologic therapies as well as for guiding junior CM practitioners, supplementing Western medicine interventions, and managing Chinese herb reserves in clinics. However, the barriers include the lack of a specialized platform for timely information release, defective plan content, limited reference value to experienced CM practitioners, and lack of local applicability to Hong Kong. The CM practitioners’ expectations on the improvement of the plans are based on the identified barriers.

The findings of this study reflect the characteristics of TCM diagnosis and treatment as well as the situation of the plan application in Hong Kong, which are in line with the findings of previous studies. Many clinical trials have suggested the beneficial effects of TCM interventions on chronic diseases or conditions, such as knee osteoarthritis [25], frozen shoulder [26], diabetes [27], asthma [28], and insomnia [29], especially in older adults. Several studies also examined and supported the effects and feasibility of self-administered TCM interventions, such as self-acupressure [30], self-moxibustion [31], parent-administered *tui na* [32], *qigong* [33], and TTCM medicinal cuisines [34]. A review on TCM medicinal cuisine and herbal medicine for COVID-19 prevention reported evidence supporting the potential antiviral ability of foods and herbs against SARS-CoV-2 and that foods and herbs can be used as TCM medicinal cuisine or complementary therapy to prevent infection and strengthen immunity [35]. Previous studies also supported the combination of TCM intervention and Western medicine. A systematic review with a meta-analysis on the use of Chinese herbs for COVID-19, involving 732 participants, reported that the combination of Chinese herbs and standard care has a superior effect on changes in symptoms and the sign core (− 1.30 by SMD, 95% CI [− 2.43, − 0.16]; three studies; *n* = 261, *P* = 0.03) and inflammatory marker C-reactive protein (CRP, mg/L; −11.82 by MD, 95% CI [− 17.95, − 5.69]) and suggested that Chinese herbs, as an adjunct treatment, with standard care can help improve treatment outcomes in COVID-19 cases [36]. Another systematic review that involved 19 clinical trials and 1,474 participants obtained similar results and concluded that the treatment of COVID-19 with CM and Western medicine may be effective in controlling symptoms and reducing the disease progression rate [37].

In this study, online focus group interviews are conducted because of the COVID-19 pandemic. Although some researchers believe that online methods are far from the traditional notion of face-to-face interaction, online moderators who facilitate the focus group discussion have no active role, and such methods lack participant engagement and a sense of response immediacy [38, 39], the online method used in this study made the data collection easy and safe for the CM practitioners and research team during the pandemic. Some researchers believe that Internet-assisted methods can offer effective means for capturing the essential elements in a focus group. Moreover, some studies suggested that the online environment may reduce the inhibitions of focus group participants and facilitate free-flowing discussions [40, 41]. A study comparing face-to-face focus group and online focus group discussions via 16 interview sessions (a total of 48 participants) found that the online focus group communication generated a high volume of ideas and solutions [42]. In our previous study, we also conducted online focus group interviews for qualitative data collection and examined their feasibility, convenience, and safety during the pandemic [43]. This strategy can enable the participation of an adequate number of CM practitioners, and the interviews were conducted in a timely manner. Although limitations associated with online focus group discussions exist, the effectiveness of qualitative data collection in this research and in previous studies is certain [44]. Therefore, in situations where face-toface focus group interviews are inconvenient or impractical due to certain reasons, real-time online methods can serve as promising alternatives for qualitative researchers.

For the issue of plan release and information sharing, though various channels are available to the practitioners for obtaining relevant information, such as TCM groups, social media, and professional training, the absence of an official platform or institution dedicated to the dissemination of TCM information in Hong Kong poses a significant challenge. This deficiency in the infrastructure may result in CM practitioners missing important guidance documents. The establishment of a platform would provide practitioners with a centralized and standardized resource hub and ensure the accessibility of essential guidance documents. For example, in Mainland China, the most important guidance plans are released on the official website of the central government [11]. Although several TCM platforms exist, such as the Chinese Medicine Council of Hong Kong [45], Hong Kong Chinese Medicine Development Fund Resource Platform [46], and GovHK Chinese Medicine Sect. [47], no official platform exists for TCM information releasing, sharing, and exchanging [48]. Thus, the creation of an electronic platform is encouraged for the release of official CM instructions or for the exchange of clinical information among CM practitioners in an effective and timely manner.

Plan localization was mentioned repeatedly by several CM practitioners. The limitations of the practicability of the plans in Hong Kong are reflected in three aspects. First, the dampness–heat pattern is the most common pattern in Hong Kong owing to its geographical location and weather [49,50]; however, the plans do not address the diagnosis and treatment of dampness–heat patterns. Second, the vast majority of patients who visit CM clinics in Hong Kong are outpatients, which differs from the mainland where the plans are more convenient for inpatients. In the future, the first CM hospital in Hong Kong will accept outpatients and inpatients [51], which may enhance the practicability of the CM plans in Hong Kong. Third, patent CMs as well as some Chinese herbs or materials for TCM medicinal cuisine therapy are not commonly used in Hong Kong owing to regulations and other factors, making their purchase difficult. Based on different situations, CM practitioners suggested supplementing the plans and allowing local CM experts to participate in their drafting or adjustment. Local CM researchers and clinical practitioners should cooperate closely to give professional and timely advice for the guidance documents during the pandemic to enhance their suitability for local use.

The research findings have several implications. For CM practitioners, this study highlights the value of the CM medicinal cuisine and non-pharmacologic therapies, to CM practitioners, especially those who are junior, to provide a more holistic care to the patients in clinical practice. For policymakers, this work identifies the merits and limitations of the current guidelines, which will inform the preparation of similar guidelines in the future. Hong Kong CM practitioners and researchers are encouraged to participate in the writing of the plans for localization. Our findings also reveal CM pracitioners’ opinions on the need for a specialized platform in Hong Kong for timely information release and experience sharing among CM practitioners and researchers. For Hong Kong citizens, this study points out that some of the suggested interventions are not available to the public, such as respiratory therapy, patent CMs, and TCM medicinal cuisine materials. Hence, the content can be further adapted to fit the local context.

### Limitations

This focus group interview study has several limitations. First, we did not conduct a pilot test to examine the feasibility of the study design, which may be useful for adjusting the methods for the research questions. Second, in online focus groups, moderators may miss the participants’ body language, which may provide information on how the participants feel about a question. Third, the moderator was acquainted with the participants, and the participants were acquainted with one another owing to their occupation, which may have affected how the participants responded to the questions and interacted with one another.

## Conclusions

The facilitators of the application of the plans included a variety of information sources, the feasibility of TCM medicinal cuisine and non-pharmacologic therapies, and the plans’ value for leading junior CM practitioners, supplementing Western medicine interventions, and guiding Chinese herb reserves in clinics. The barriers to the application of the plans included the lack of a specialized CM platform for information release and exchange, defective plan content, and lack of localization in Hong Kong. Accordingly, the CM practitioners expected to have a specific CM platform and to perfect the plans to make them feasible, targeted, thorough, and localized. To enhance the implementation of the anti-epidemic plans, the CM practitioners from Hong Kong expected to utilize a specific CM platform and refine the plans to ensure that they are realistic, focused, comprehensive, and tailored to the local context.

### Electronic supplementary material

Below is the link to the electronic supplementary material.

Supplementary Material 1

## Data Availability

The datasets used and/or analysed during the current study are available from the corresponding author on reasonable request.

## Abbreviations

TCM: traditional Chinese medicine
CM: Chinese medicine
