# Supplementary material for: Views of Hong Kong Chinese medicine practitioners on the application of the “Chinese Medicine Anti-epidemic Plans” prepared by the Chinese medicine expert group of central authorities: a focus group study
Source: BMC Complement Med Ther. 2024 May 4;24:184. doi: 10.1186/s12906-024-04469-3 (PMC11069236; doi:10.1186/s12906-024-04469-3)
Supplement: Supplementary file 1 — Supplementary Material 1 [file 12906_2024_4469_MOESM1_ESM.docx]

**Appendix 2: Coding tree and quotation**

|  | Sub-themes | Code Units |
| --- | --- | --- |
| - Facilitators of the “Chinese Medicine Anti-epidemic Plans” | - Diversified information channels | - **Traditional Chinese medicine groups, social media, work, non-profit organizations, hospital authority, training, and reference to the mainland version**   “Some TCM groups, or classmate groups shared with everyone. (P1)”  “I remember I hearing about that on TV news, and I had seen it in the association. (P2)” |
|  | - Reference value of the plans | - **TCM medicinal cuisine, acupuncture and moxibustion, non-pharmacological interventions, and others have a reference value.**   “Rehabilitation guidelines have their benefits, such as TCM medicinal cuisine, If patients have any inquiries, then you can answer them by referring to the guidelines. **(P11)”**  “Acupuncture and moxibustion interventions are described in detail instead, specific acupuncture points and duration are covered, massage therapy techniques are also included **(P5)**” |
|  | - The value to patients | - **It holds reference value for the application in the Asia World-Expo Isolation ward (Asia World-Expo)**   “It was used frequently in AsiaWorld-Expo**.** (P15)”   - **Effective for inpatients, patients with comorbid chronic diseases, and patients with deficiency syndromes**   “They are sent from hospitals to AsiaWorld-Expo; most patients are old, with chronic diseases and deficiency syndromes… I think that the prescription is really useful for these elderly people. They don’t cough much and their symptoms are related to comorbid chronic diseases... Most cases have recovered from illness after taking traditional Chinese medicines. (P16)” |
|  | - The value to TCM practitioners | - **The guideline is useful for TCM practitioner with limited clinical experience**   “Fresh graduate TCM practitioners believe it has reference value, while highly experienced ones think it has none. **(P21)**” |
|  |  | - **Prepare more TCM resources for practitioners address the demand and complement Western medicine therapy**   “…(For some symptoms during COVID-19), western medicine can’t do much, there were no Covid-19 antivirals at that time…there is really no medicine to treat him. Just take these traditional Chinese medicines. (P16)” |
|  |  | - **During the epidemic, it can be used diagnosis to guide the reserve of traditional Chinese medicine and prescription ideas.**   “After looking through this material, I may remind myself that such people (patients) exist... I may not have made a prescription of pure ginseng decoction before. Should I prepare some *Codonopsis pilosula* (similar effect to ginseng) in the clinic first? Compared to the one released by the National Health Commission, this (Hong Kong) version may provide more insight in these areas **(P7)”** |
|  |  |  |
| - Barriers of the “Chinese Medicine Anti-epidemic Plans” | - Issues on plan release | - **No official release channel, difficult to search, and uncertain version accuracy**   “I know from the news that there would be a plan, but it is difficult to search, no matter which channel you used. We currently do not have an official channel for traditional Chinese medicine. **(P7)**” |
|  |  | - **Released too late, pass the peak of the epidemic.**   “I feel that if it was released earlier, with this version, I could accept it. Because at that time, when everyone started their clinical work, we have no chance to see these patients. This plan tells us what we will encounter, so it is all right. However, it was released in April, and the peak was over. (**P15)”** |
|  | - Defects in the content of the plans | - **Confusion in the management of prevention/treatment/prognosis, incomplete content**   “I don’t know why in the industry there is often confusion between prevention, treatment and post-treatment. Why does one prescription, initially meant for prevention, turn to that for post-treatment? I think this is a big issue **(P17)”** |
|  |  | - **The treatment methods have limitations:** **Collecting the ingredients for TCM medicinal cuisine is difficult, and the feasibility of non-pharmacological interventions (e.g., acupuncture and moxibustion, massage, TCM medicinal cuisine, emotional therapy and respiratory therapy) is poor.**   “In addition, they (the suggested plans) are very complicated. *Siraitia grosvenorii*, *Ficus carica*, Asian pear, orange, radish, and olives should be all included..., which is difficult. **(P12)”**  “Some of them are complicated. I won’t do moxibustion and massage, so I won’t refer to the plan. **(P12)”** |
|  |  | - **Limited use for outpatient clients and sequelae**   “Many patients who suffered from COVID-19 have different diseases (symptoms), these Chinese medicine prescriptions are useless, for those with emotional problems, these prescriptions may not help. **(P16)”** |
|  | - Low reference value to TCM practitioners | - **When it was released, TCM practitioners had accumulated experience and had not fully agreed with the prescriptions in the plan.**   “After the clinical guidelines were released, we had already conduct consultations with a lot of patients, the variant was Omicron, and we had basically mastered the entire pattern. So, the subsequent information didn't hold much reference value for us. **(P1)”** |
|  | - Unsuitable for local application | - **Lack of local typical syndrome types in the plan (e.g., damp-heat syndrome)**   “It does not cover the therapy for the late-stage damp-heat disease and warm-heat disease. There is no content related to the warm-heat disease. **(P12)”** |
|  |  | - **Majority of the local cases involve outpatient visits**   “I understand all the contents in the COVID-19 guidelines, but it is difficult to implement in Hong Kong due to insufficient conditions. Most of the cases we have are outpatient visits. **(P9)” (outpatients visits refer to patients who are not isolated in facilities such as the AsiaWorld-Expo or Mobile Cabin Facilities.)** |
|  |  |  |
| - Expectations on improving the “Chinese Medicine Anti-epidemic Plans” | - Plan release mechanism improvement | - **Suggested establishing an official organization for disseminating information.**   “In mainland China, the National Health Commission and State Administration of Traditional Chinese Medicine are responsible for jointly organizing experts to formulate these diagnosis and treatment plans, which are then publicly released. Therefore, a similar institution should be established in Hong Kong. **(P22)”** |
|  |  | - **A Cantonese version is needed.**   “For respiratory therapy, shall it be practice using Mandarin pronunciation? If it is pronounces in Mandarin, then it is different from our Cantonese pronunciation. Thus, do we need to make a Cantonese version? **(P18)”** |
|  |  | - **Provide forward-looking information and continuously update the plans**   “There should be an organization responsible for updating the information for doctors regularly (e.g., quarterly updates). **(P15)”** |
|  | - Plan content improvement | - **Supplement diagnosis and treatment contents (etiology and pathogenesis, diagnose and medication, provide detailed acupuncture and moxibustion protocol, clear visual teaching of Qigong and other therapies)**   “I would like to know how those symptoms differ from the symptoms I usually see, or how they differ from the common cold symptoms I have seen before. **(P3)”**  “Pathogenesis, disease mechanisms, and identifying high-risk groups, so that doctors know which are critical symptoms and how to handle them accordingly.". **(P22)”**  “Add more content about Western medicine, including the intervention times of TCM. At that time, I could imagine that the combination of Chinese and Western medicines would be even better. This plan then will have a great reference value, such as what kind of dialectics and what medicine to use. **(P5)”**  “For acupuncture, there is no distinction between main acupoints, and there is no selection of acupoints based on syndrome differentiation. I believe that it can be written in more detail, and there are some aspects that I don’t know why they do it in this way. **(P5)”** |
|  |  | - **Provide more targeted medication recommendations based on known clinical data**   “Write down how to use Western and traditional Chinese medicine in parallel, and then observe the clinical performance of patients… in this way, there is a clinical reference value. Medication treatment should be more clear, precise, and targeted … We cannot always generalize…. **(P9)”**  “If a plan is issued, it may need to be divided into different directions. One is targeting the community, which can be accessed earlier. The other one is for various centers (isolation facilities). **(P22)”** |
|  |  | - **Supplement information sources**   “The clinical protocol should have a references. If there are reference, everyone can look them up for further review. Maybe this could be much better. **(P8)”** |
|  |  | - **Summarizing and sharing of clinical cases**   “I think sharing actual cases ..has more practical utility ...and, without pulse taking and tongue inspection, how they prescribe medication and diagnose. I think these experiences are useful, but it may be challenging to incorporate them into the guidelines. **(P2)”** |
|  | - Enrichment of information sources for plan development | - **Organize local TCM practitioners to rapidly gather diagnosis and treatment experience and characteristics of the disease during the outbreak**   “Because nobody knows the characteristics and pattern of the disease in the human body, everyone is exploring. If you have the information, write it down. **(P1)** |
|  |  | - **Establish a communication platform to collect all information and opinions**   “Experience can be presented on a platform. Those who have less experience can gradually learn from more experienced TCM practitioners. In this way, when others are working, there will be new experience. This will not only help quickly establish a systematic framework, but it also adds reference value. **(P5)”** |
|  |  | - **The framework of the plan should be based on the clinical treatment experience, and reference to expert suggestions to enhance the treatment approach.**   “Different schools of thought, for example, those who use classical prescriptions, may have a different perspective from other TCM practitioners. There will be more diverse reference ideas and elements. I think this is better when compared to the current textbook-style or syndrome-based approach. (**P4)”** |
|  | - Plan localization | - **Tailor treatment according to three categories of etiological factors, refining the syndrome differentiation, diagnosis and treatment content to adapt to the local climate and seasonal variations**   “I believe that for the same viral pathogen, there should be variations bases on different seasons and weather conditions . **(P17)”** |
|  |  | - **Involve local TCM teams in formulating the plan.**   “You need frontline TCM experts from our region... You definitely won't have enough understanding of our situation compared to local TCM practitioners who have been practicing here for over a decade. **(P15)”** |
